# Air Pollution Impairs Marathon Performance: A Cross-Sectional Analysis of 2.7 Million Finishers Across Six World Marathon Majors

**DOI:** 10.1186/s40798-026-01099-6

**Published:** 2026-08-29

**Authors:** James A. Donaldson, Samuel Cai, Josh Vande Hey, Anna L Hansell, Thomas Yates, G. Andre Ng, Jamie O’Driscoll

**Affiliations:** 1 Division of Cardiovascular Sciences, University of Leicester, Leicester, UK; 2 Division of Public Health and Epidemiology, School of Medical Sciences, Centre for Environmental Health and Sustainability, University of Leicester, Leicester, UK; 3 NIHR Leicester Biomedical Research Centre, Leicester, UK; 4 Leicester British Heart Foundation Centre of Research Excellence, University of Leicester British Heart Foundation Cardiovascular Research Centre, Glenfield Hospital, Groby Road, Leicester, UK; 5 NIHR Health Protection Research Unit in Chemical Threats and Hazards, Leicester, UK; 6 Diabetes Research Centre, College of Life Sciences, University of Leicester, Leicester, UK; 7 Leicester General Hospital, Leicester Diabetes Centre, University Hospitals of Leicester NHS Trust, Leicester, UK

**Keywords:** Air pollution, Nitrogen dioxide, Particulate matter, Running, Athletic performance, Environmental exposure, Exercise

## Abstract

**Background:**

Ambient air pollution impairs respiratory and cardiovascular function during exercise, yet evidence on its effects across the full spectrum of endurance performance remains limited.

**Objectives:**

To quantify the association between race-day ambient air pollution and marathon finishing time across six World Marathon Majors, and to determine whether effects vary by sex, age group, and performance level.

**Methods:**

We analysed 2,756,553 net finishing times from six World Marathon Majors (Berlin, Boston, Chicago, London, New York City, and Tokyo) for editions held between 2010 and 2024. Race-day nitrogen dioxide (NO₂) and fine particulate matter (PM₂.₅) concentrations were obtained from the Copernicus Atmosphere Monitoring Service (CAMS) global reanalysis dataset. Linear mixed-effects models with random intercepts for each of 75 marathon–year events estimated pollution effects on finishing time, adjusting for heat index, wind speed, and temporal trends. Stratified analyses were conducted among 1,815,188 runners with available age data.

**Results:**

Of the pollutants examined, NO₂ showed the stronger association with finishing time. Each 1 µg/m^3^ increase was associated with 1.43 min slower finishing time in men (95% confidence interval [CI] 0.66 to 2.21) and 1.56 min in women (95% CI 0.63 to 2.48). PM₂.₅ effects were not statistically significant: −0.13 min per µg/m^3^ in men (95% CI −0.67 to 0.40) and −0.14 min per µg/m^3^ in women (95% CI −0.76 to 0.49). Recreational runners experienced NO₂ effects approximately six-fold larger than elite runners (2.12 vs 0.34 min per µg/m^3^); expressed as a percentage of mean finishing time, this gradient persisted (0.67% vs 0.18%). Younger runners (18–29 years) showed larger effects than older runners (2.17 vs 1.32 min per µg/m^3^ for ≥60 years). Kolmogorov–Smirnov tests confirmed that higher NO₂ was associated with rightward shifts across the entire finishing time distribution.

**Conclusions:**

Ambient air pollution, particularly NO₂, is associated with impaired marathon performance in a dose-dependent manner, with the largest effects among recreational runners. These findings have implications for race management and public health policy regarding outdoor physical activity in urban environments.

**Supplementary Information:**

The online version contains supplementary material available at https://doi.org/10.1186/s40798-026-01099-6.

## Keypoints

- Race-day NO₂ exposure was associated with substantially slower marathon finishing times across 2.7 million performances at the six World Marathon Majors, while PM₂.₅ effects were not statistically significant after adjustment for weather.
- In over 2.7 million marathon performances across six World Marathon Majors, race-day NO₂ was associated with substantially slower finishing times, with recreational runners experiencing approximately six-fold greater performance impairment than elite runners. Pollution shifted the entire finishing time distribution, not solely mean performance.
- Air quality and event-related traffic policies should be considered alongside temperature and humidity when planning urban endurance events and advising runners, particularly recreational participants who may be most vulnerable to pollution-related performance impairment.

**Supplementary Information:**

The online version contains supplementary material available at https://doi.org/10.1186/s40798-026-01099-6.

## Introduction

Athletes exercising in urban environments may be particularly vulnerable to environmental exposures that impair respiratory and cardiovascular function. During endurance exercise, increased minute ventilation and predominant oral breathing reduce nasal filtration and increase pollutant deposition in the lower respiratory tract [1]. Ambient pollutants, such as nitrogen dioxide (NO₂) and particulate matter (fine particulate matter, PM_2.5_; and PM_10_) have been linked to impaired lung function, airway inflammation, endothelial dysfunction, and adverse cardiovascular responses [2–5]. Experimental studies have demonstrated that acute exposure to NO₂ and particulate matter can reduce exercise performance through mechanisms including oxidative stress, vasoconstriction, and inflammatory responses [6, 7].

Observational studies of endurance events have reported associations between pollution exposure and slower race performance [8–12]. However, important gaps remain. First, prior analysis have either focused on elite athletes (e.g., podium finishers) [12], or reported mean effects across entire fields [8, 10], limiting insight into heterogeneity by age, sex and performance level. Second, most studies have examined average or percentile specific effects without formally evaluating whether pollution shifts the entire distribution of finishing times [9]. Third, weather conditions, particularly temperature and humidity, strongly influence marathon performance [13, 14] and correlate with pollution levels, yet their role in distributional analyses has not been adequately addressed.

Major international marathons provide a unique natural experiment to examine pollution–performance relationships across diverse environmental contexts. These races occur in different cities and seasons, encompass a wide spectrum of athletic ability, and generate standardised performance data for large populations. Understanding whether pollution disproportionately affects recreational versus elite runners has important implications not only for competitive sport, but also for public health, given the growing participation of older and non-elite individuals in endurance events [15].

This study aimed to quantify the association between race-day ambient air pollution and marathon performance across the six World Marathon Majors in a large, multinational cohort of more than 2 million finishers, and to determine whether this relationship varies by sex, age group and performance level, including shifts across the full distribution of finishing times.

## Methods

### Data Sources

#### Marathon Performance Data

We obtained individual-level marathon results from the official results websites of the six World Marathon Majors (Berlin, Boston, Chicago, London, New York City, and Tokyo) for editions held between 2010 and 2024 using custom web-scraping scripts written in Python. Table 1 summarises the characteristics of each marathon. Not every city was held, or had publicly available individual results, in every year of this window: the 2020 editions were cancelled or held virtually because of the COVID-19 pandemic, the 2012 New York City Marathon was cancelled in the aftermath of Hurricane Sandy, the Tokyo Marathon is included from 2015 onward (when comparable individual results became available), and a small number of further city-years were unavailable. This yielded 75 marathon–year events in total (Table 1).

**Table 1:** Characteristics of the six world marathon majors included in the analysis (2010–2024, excluding 2020)

Marathon	Years	N Events	N Runners	% Female	NO₂ (µg/m^3^)	PM₂.₅ (µg/m^3^)	Temperature (°C)	Humidity (%)	Wind speed (m/s)	Median finish time (min)
Berlin	2010–2024	14	517,647	27.8	6.1 (5.0–7.6)	9.9 (8.7–13.9)	14.8 ± 2.5	72.4 ± 10.1	10.8 ± 3.7	244 (217–280)
Boston	2010–2024	14	327,462	43.9	6.6 (4.7–9.6)	14.1 (8.9–20.3)	13.0 ± 4.9	65.7 ± 17.9	17.0 ± 6.5	227 (203–260)
Chicago	2010–2024	13	517,212	46.0	7.7 (5.5–8.9)	5.5 (1.1–12.5)	14.5 ± 4.4	63.9 ± 10.9	17.9 ± 5.2	267 (230–310)
London	2010–2024	14	538,442	38.9	10.9 (8.7–14.5)	12.5 (8.4–20.1)	11.8 ± 3.1	67.4 ± 6.9	14.8 ± 4.9	267 (229–309)
New York City	2010–2024	13	614,942	41.2	7.3 (6.3–11.5)	3.7 (1.7–7.7)	10.7 ± 4.0	62.3 ± 12.0	13.7 ± 6.1	269 (236–308)
Tokyo	2015–2024	7	241,643	22.9	8.6 (4.8–12.9)	25.6 (17.7–32.0)	7.1 ± 1.4	63.6 ± 10.9	43.1 ± 12.2	288 (238–339)
**Total/overall**	**2010–2024**	**75**	**2,757,348**	**37.8**	**7.5 (5.2–11.1)**	**10.4 (6.2–17.8)**	**12.3 ± 4.3**	**66.0 ± 12.0**	**17.1 ± 10.5**	**259 (224–301)**

*NO₂* nitrogen dioxide, *PM₂.₅* fine particulate matter (< 2.5 µm). Pollution values are event-level summaries derived from the CAMS global reanalysis (EAC4), averaged over the daytime race window (approximately 06:00–18:00 local time). Summary statistics shown as median (IQR) for pollution and finish time, mean ± SD for meteorological variables. The 2020 editions (cancelled or virtual owing to COVID-19), the 2012 New York City Marathon (cancelled after Hurricane Sandy), and a small number of further unavailable city-years are not represented; Tokyo is included from 2015

We validated the scraped data in two ways. First, we performed manual spot-checks in which random samples of scraped records, together with the published leading finishers for each race-year, were cross-checked against the original official results pages to confirm that names, finishing places and finishing times matched. Second, we applied automated consistency checks, verifying that finisher counts and the ordering of finishing times by overall finishing place were internally consistent for each event. Finishing times were then restricted to 120–480 min to exclude implausible values and recording errors; the lower bound approximates the men’s marathon world record, and the upper bound reflects typical course time limits imposed by race organisers. After applying these criteria and excluding the 2020 London Marathon (held as a closed elite-only/virtual event because of COVID-19), the final analytical sample comprised 2,757,348 marathon performances across 75 marathon–year events. Of the full set of finishers, 795 records could not be assigned a male or female classification from the official race data and were excluded from all sex-stratified models, leaving a final analytical sample of 2,756,553 performances (males: 1,713,151 [62.1%]; females: 1,043,402 [37.9%]).

One race-year, Boston 2013, was retained in the primary analysis but examined separately in a sensitivity analysis: the 2013 Boston Marathon was halted before all participants finished, and although the times we obtained correspond only to runners who completed the course before it was closed (and do not include any projected times subsequently issued to stopped runners), the resulting truncation of the slower portion of the field makes this race-year potentially unrepresentative(see: Sensitivity Analyses; Supplementary Table S5).

#### Air Pollution Data

Race-day air pollution concentrations were obtained from the Copernicus Atmosphere Monitoring Service (CAMS) global reanalysis (EAC4) dataset [16] which provides three-hourly gridded estimates of atmospheric composition at 0.75° × 0.75° spatial resolution. For this study, concentrations of fine particulate matter (PM₂.₅) and nitrogen dioxide (NO₂), expressed in μg/m^3^, were extracted at the geographic coordinates corresponding to each race start location using point extraction from the nearest grid cell (i.e. not spatially weighted along the race route). Pollution exposure was assigned at the marathon–year event level; thus, all participants within a given event were assigned the same race-day concentration.

Race-day mean pollutant concentrations were calculated by averaging the three-hourly CAMS values falling within the daytime race window from 6:00 AM to 6:00 PM local time at each location, which spans official race start (typically 7:00–9:00 AM local). To validate these reanalysis-based estimates, we compared the CAMS race-window values against contemporaneous measurements from ground-level monitoring networks in five of the six cities (US EPA Air Quality System for Boston, Chicago and New York City; UK DEFRA Automatic Urban and Rural Network for London; and the Berlin Luftgütemessnetz for Berlin; comparable public data were not available for Tokyo); the results of this validation are summarised in the Sensitivity Analyses and reported in full in the Supplementary Material (Ground Monitor Validation). Sensitivity analyses also examined alternative exposure-averaging windows and definitions to assess the robustness of results to exposure timing assumptions.

#### Meteorological Data and Heat Index

Weather information was retrieved from the Open-Meteo Historical Weather API [17], which provides ERA5-based reanalysis data interpolated to surface level. For each race day, measurements of 2-m above-ground temperature (°C), relative humidity (%), and wind speed (m/s) were extracted at the race start coordinates and averaged over the same 6:00 AM to 6:00 PM local time window as used for pollution exposure.

We calculated heat index using the NOAA Rothfusz regression equation [9], which combines temperature and relative humidity into a single measure of apparent temperature that reflects the physiological experience of heat stress. This approach has been widely used in studies of environmental effects on athletic performance.

### Variable Construction

#### Outcome Variable

The primary outcome was finishing time in minutes, derived from chip-timed individual results. All six marathon series report net finishing times on their official results platforms, and these net times were the values extracted by our scrapers; the timing basis for each series is documented in the Supplementary Material.

#### Demographic Variables

Age groups were defined using standard masters running categories commonly employed in competitive racing: 18–29, 30–39, 40–49, 50–59, and ≥60 years. Sex was coded as male (M, *n*=1,713,151) or female (F, *n*=1,043,402) based on official race records; 795 records that could not be classified were excluded from all sex-stratified models (see Marathon Performance Data above).

#### Performance Level Classification

To assess heterogeneous effects across runner ability, we classified runners into six performance levels based on within-race percentile rankings. For each marathon–year combination, we calculated each runner’s percentile rank (0 = fastest finisher, 100 = slowest finisher) and assigned them to performance categories: elite, sub-elite, upper mid-pack, mid-pack, recreational, back of pack (see sample size and finishing time for each category in Supplementary Table S2). This within-race ranking approach, adapted from percentile-based classifications used in prior marathon research (El Helou et al., 2012; Lepers & Cattagni, 2012), accounts for differences in course difficulty, field competitiveness and weather across events.

### Statistical Analysis

#### Descriptive Statistics

We calculated summary statistics for finishing times, pollution concentrations, and weather conditions overall and stratified by marathon and year. Pollution–weather correlations were assessed using Pearson correlation coefficients.

#### Linear Mixed-Effects Models

Our primary analytical approach employed linear mixed-effects models (LMMs) to estimate the association between race-day air pollution and marathon finishing time. LMMs account for the nested structure of our data (individual runners within marathon–year events) and between-event heterogeneity in baseline finishing times due to unmeasured factors such as course characteristics and field composition.

The model for individual *i* in marathon–year event *j* was specified as:

$$ Finish\,Time\, = \,\beta_{0} \, + \,\beta_{1} \,Pollua\tan t_{j} \, + \,\beta_{2} \,Year_{j} \, + \,f\left( {HeatIndex_{j} } \right) + \,\beta_{3} Wind_{j} \, + \,u_{j} + \varepsilon_{ij} , $$

where Pollutantj is the race-day mean concentration of NO₂ or PM₂.₅ (µg/m^3^) entered separately in single-pollutant models, f(HeatIndexj) is a natural cubic spline (5 degrees of freedom), Yearj is a linear trend, Windj is the race-day mean wind speed in m/s, uj ~ N(0, σ^2^u) is a random intercept for each marathon–year event, and εij is the residual error.

Since pollution exposure varies at the marathon–year event level, effect estimates are identified from between-event variation across marathon–year combinations. Air pollution was analysed as a continuous exposure variable. All primary models were estimated separately in men and women (sex-stratified), and separately for each pollutant. Models were estimated using restricted maximum likelihood in the lme4 package in R, with Satterthwaite degrees of freedom (lmerTest) for p-values; 95% confidence intervals for the pollutant coefficient were computed as the estimate ± 1.96 × standard error. Model assumptions were assessed using residual and Q–Q plots. Statistical analysis and presentation were reviewed for consistency with the CHAMP statement [18].

#### Stratified Analyses for Effect Heterogeneity

To assess whether pollution effects vary by runner characteristics, we estimated separate LMMs stratified by performance level (six categories: elite through back of pack), age group (18–29, 30–39, 40–49, 50–59, and ≥60 years), and cross-classified age × performance strata (up to 30 combinations, strata with N<500 were excluded). For analysis involving age, the sample was restricted to runners with available age data (*N*=1,815,188). Stratified models used the same specification as the primary model and were fitted separately within each stratum.

#### Distributional Analysis: Kolmogorov–Smirnov Tests

To complement mixed-effects models of conditional mean differences, we conducted two-sample Kolmogorov–Smirnov (K-S) tests to assess whether pollution was associated with shifts across the full distribution of finishing times. High and low pollution days were defined at the marathon–year event level using the >75th percentile and <25th percentiles of race-day pollutant concentrations across all events. Weather-residualised K-S tests were performed using a two-stage procedure: (1) regress finishing time on heat index and wind speed within each marathon–year event to obtain weather-adjusted residuals; (2) compare the distribution of residuals between high- and low-pollution events. Residualised K-S tests were conducted overall, within individual marathons, and stratified by performance level. Supplementary Table S1 details the within-marathon estimated finishing time differences between high and low pollution days.

#### Sensitivity Analyses

We conducted several sensitivity analyses to assess robustness, including alternative heat index specifications (quadratic terms and natural splines with 3–7 degrees of freedom), exclusion of extreme pollution events (> 95th percentile or <5th percentile), alternative exposure windows aligned with race start and median finish time, exclusion of the 2013 Boston Marathon (which was halted before all participants finished; see Methods), and exclusion of all Boston Marathon editions (2010–2024) to address potentially weaker CAMS PM₂.₅ agreement at Boston (ground-monitor Pearson r = 0.09). Additionally, two-pollutant models including both NO₂ and PM₂.₅ simultaneously were fitted to assess mutual confounding and effect attenuation. The significance of the random intercept was evaluated using a likelihood ratio test comparing mixed and fixed-effects specifications. To complement the absolute effect estimates (in minutes), we additionally expressed each NO₂ effect as a percentage of the relevant subgroup’s mean finishing time, to assess whether heterogeneity across sex, age and performance level reflected differences in proportional rather than absolute susceptibility.

### Software and Reproducibility

Analyses were performed in Python 3.12 and R 4.3. The complete codebase and processed datasets are available online (https://github.com/jamesad90/marathon-pollution).

### Patient and Public Involvement

No patients or members of the public were involved in the design, conduct, or reporting of this research.

### Equity, Diversity, and Inclusion

This study incorporated data from six World Marathon Majors spanning three continents (Europe, North America, and Asia), encompassing both male and female runners across a wide age range (18 to >60 years) and the full spectrum of performance levels from elite to recreational. The research team includes members of multiple career stages and research disciplines.

### Ethical Considerations

Ethical approval was not required because the study used publicly available, anonymised data and did not involve human participants, their data, or biological material requiring ethics approval.

## Results

### Study Sample

**Fig. 1:**
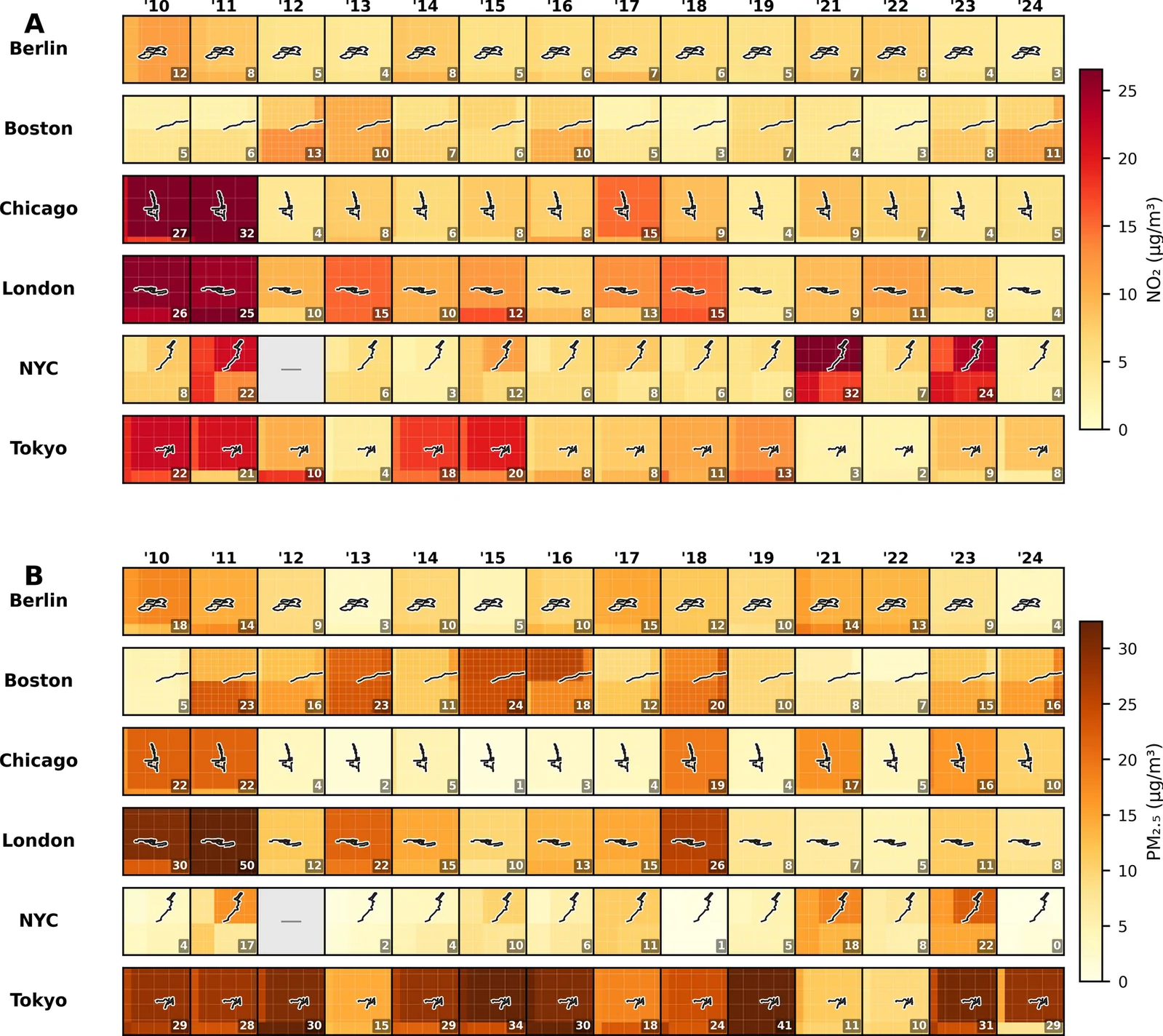
Race-day air pollution concentrations across six World Marathon Majors, 2010–2024. (A) NO₂ and (B) PM₂.₅ concentrations (µg/m³) by marathon and year. Each cell represents one race-year event and shows the marathon location or course in relation to the corresponding CAMS model grid cell from which the race-day pollution concentration was extracted. The outlined line within each cell represents the marathon course where route data were available; for Berlin and Tokyo, the race location is shown schematically. The background shading represents the spatial distribution of pollutant concentrations within and around the relevant CAMS grid cell, while the number in the lower-right corner gives the race-day concentration assigned to that event. The absence of 2020 events reflects cancellations due to the COVID-19 pandemic; the 2012 New York City Marathon was cancelled following Hurricane Sandy. NO₂, nitrogen dioxide; PM₂.₅, fine particulate matter; CAMS, Copernicus Atmosphere Monitoring Service

A total of 2,756,553 marathon performances across 75 marathon–year events from the six World Marathon Majors (2010–2024) were included in the final analytical sample (2,757,348 total performances after time-window filtering, less 795 records that could not be assigned a sex classification). Of these, 1,713,151 (62.1%) were male and 1,043,402 (37.9%) were female (795 performances without a male/female classification in the official records were excluded from the sex-stratified models). Race-day NO₂ concentrations ranged from approximately 2.8 to 32.3 µg/m^3^ (interquartile range [IQR] 5.1 µg/m^3^) and PM₂.₅ from approximately 0 to 50.3 µg/m^3^ (IQR 11.0 µg/m^3^) across events (Fig. 1).

### Association Between Air Pollution and Finish Time

**Fig. 2:**
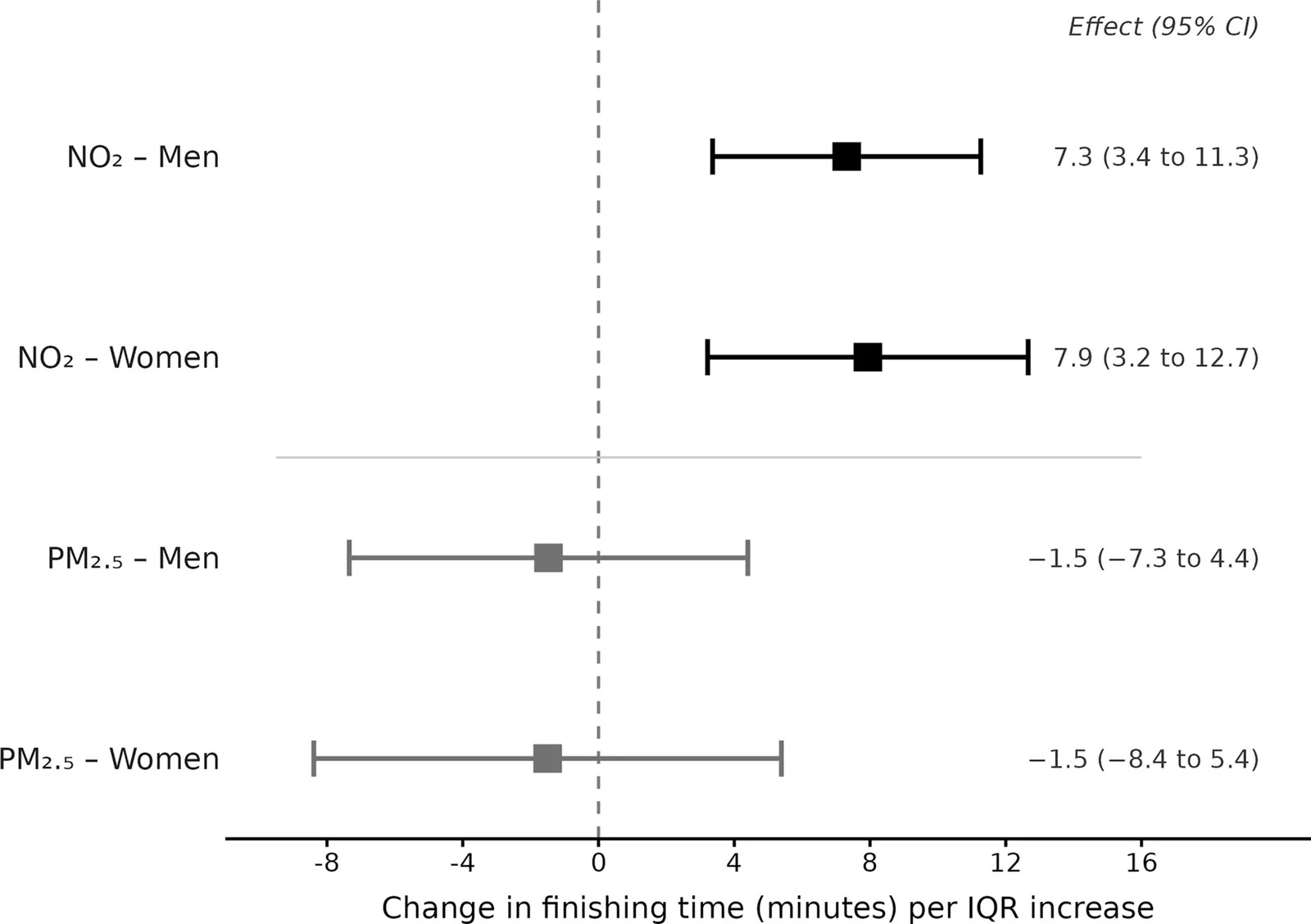
Forest plot of air pollution interquartile range (IQR) effects on marathon finishing time by sex. Points show the estimated change in finishing time (minutes) associated with moving from the 25th to the 75th percentile of pollutant concentration, with 95% confidence intervals, from linear mixed models. All models adjusted for heat index (natural cubic spline, 5 degrees of freedom), wind speed, and year, with random intercepts for the 75 marathon–year events, and were estimated separately in men and women. NO₂ IQR = 5.1 µg/m^3^; PM₂.₅ IQR = 11.0 µg/m^3^

Of the two pollutants examined NO₂ demonstrated the strongest and most consistent association with marathon finishing time (Fig. 2, Table 2). In adjusted linear mixed models, each 1 µg/m^3^ increase in NO₂ was associated with 1.43 min slower finish time in men (95% CI 0.66 to 2.21) and 1.56 min slower in women (95% CI 0.63 to 2.48). Correspondingly, moving from the 25th percentile (5.2 µg/m^3^) to the 75th percentile (10.3 µg/m^3^) of NO₂ exposure was associated with 7.3 min (95% CI 3.4 to 11.3) slower finishing time in men and 7.9 min (95% CI 3.2 to 12.7) in women (Table 2). Associations with PM₂.₅ were weaker and not statistically significant. Each 1 µg/m^3^ increase in PM₂.₅ was associated with − 0.13 min (i.e. a non-significant change towards faster times) in men (95% CI − 0.67 to 0.40) and −0.14 min in women (95% CI − 0.76 to 0.49). For PM₂.₅, the corresponding interquartile-range effect (moving from the 25th to 75th percentile, 4.4 to 15.5 µg/m^3^, IQR 11.0 µg/m^3^) was − 1.5 min (95% CI − 7.3 to 4.4) in men and − 1.5 min (95% CI − 8.4 to 5.4) in women — small, non-significant, and in the opposite direction to the NO₂ effect (Table 2). Although the absolute NO₂ effect appeared marginally larger in women than men (1.56 vs 1.43 min per µg/m^3^), the two estimates were near-identical when expressed as a percentage of mean finishing time (0.55% in women versus 0.56% in men), indicating that the apparent sex difference reflects women’s longer average finishing times rather than a greater proportional susceptibility to NO₂.

**Table 2:** Effects of air pollution on marathon finishing time by sex: linear mixed model results

Pollutant	Sex	Effect per µg/m^3^ (minutes) [95% CI]	Effect per IQR (minutes) [95% CI]	P-value	N runners
Single-pollutant models					
NO₂	Male	1.43 [0.66, 2.21]	7.31 [3.36, 11.26]	6.4 × 10⁻^4^	1,713,151
NO₂	Female	1.56 [0.63, 2.48]	7.94 [3.21, 12.66]	0.002	1,043,402
PM₂.₅	Male	− 0.13 [− 0.67, 0.40]	− 1.47 [− 7.35, 4.40]	0.625	1,713,151
PM₂.₅	Female	− 0.14 [− 0.76, 0.49]	− 1.50 [− 8.39, 5.39]	0.671	1,043,402
Two-pollutant models (NO₂ and PM₂.₅ simultaneously)					
NO₂	Male	1.91 [1.08, 2.73]	9.74 [5.53, 13.94]	3.3 × 10⁻^5^	1,713,151
NO₂	Female	2.06 [1.07, 3.06]	10.53 [5.44, 15.61]	1.7 × 10⁻^4^	1,043,402
PM₂.₅	Male	− 0.66 [− 1.17, − 0.15]	− 7.27 [− 12.89, − 1.65]	0.014	1,713,151
PM₂.₅	Female	− 0.71 [− 1.32, − 0.09]	− 7.75 [− 14.53, − 0.96]	0.029	1,043,402

*LMM* linear mixed model with random intercepts for marathon–year event. All models adjusted for heat index (natural cubic spline), wind speed, and year, and were estimated separately in men and women. *NO₂* nitrogen dioxide, *PM₂.₅* fine particulate matter. Effects are expressed in minutes of finishing time per 1 µg/m^3^ and per interquartile range (IQR) increase in the pollutant (NO₂ IQR = 5.10 µg/m^3^; PM₂.₅ IQR = 10.99 µg/m^3^). 95% confidence intervals are estimate ± 1.96 × standard error. Positive values indicate longer (slower) finishing times. The significant negative two-pollutant PM₂.₅ coefficient is interpreted as an artefact of co-pollutant adjustment (see Discussion*)*

### Two-Pollutant Models

In two-pollutant models including both NO₂ and PM₂.₅ simultaneously, the NO₂ coefficient increased by approximately one-third in both sexes (men: 1.91 min per µg/m^3^, 95% CI 1.08 to 2.73; women: 2.06 min per µg/m^3^, 95% CI 1.07 to 3.06), while the PM₂.₅ coefficient reversed sign and became statistically significant in the negative direction (men: −0.66 min per µg/m^3^, 95% CI − 1.17 to − 0.15; women: − 0.71 min per µg/m^3^, 95% CI − 1.32 to − 0.09). NO₂ and PM₂.₅ were only modestly correlated at the event level (Pearson r = 0.32). As discussed below, we interpret this negative PM₂.₅ coefficient as an artefact of co-pollutant adjustment rather than as evidence that PM₂.₅ improves performance; this coefficient was attenuated and lost significance in women when the 2013 Boston Marathon was excluded (Supplementary Table S5).

### Sensitivity Analyses

Results were robust across all sensitivity specifications. NO₂ coefficients remained stable under alternative heat index specifications (quadratic terms and natural splines with 3–7 degrees of freedom), exclusion of extreme pollution events (5th–95th percentile restriction), and linear heat index models (see Supplementary Table S5). Excluding the 2013 Boston Marathon, which was halted before all participants finished, did not materially change the NO₂ association (men: 1.47 min per µg/m^3^; women: 1.60 min per µg/m^3^) and left the single-pollutant PM₂.₅ association null; the negative two-pollutant PM₂.₅ coefficient was attenuated and lost statistical significance in women (Supplementary Table S5). The random intercept for marathon–year event was highly significant across all models (likelihood ratio test p < 0.001; intraclass correlation coefficient = 6.9–11.3%), confirming substantial between-event heterogeneity in baseline finishing times.

To address the potential concern surrounding whether our reported poor CAMS PM₂.₅ agreement at Boston (ground-monitor Pearson r = 0.09) may have introduced differential measurement error across events, we additionally excluded all 14 Boston Marathon editions from the primary models (61 events remaining; men N = 1,529,431, women N = 899,726). NO₂ associations remained highly statistically significant in both sexes (men: 1.01 min/μg/m^3^, 95% CI 0.62 to 1.40; women: 1.11 min/μg/m^3^, 95% CI 0.71 to 1.50). Notably, single-pollutant PM₂.₅ estimates shifted to small positive values (men: 0.29 min/μg/m^3^, 95% CI 0.00 to 0.58, p=0.05; women: 0.38 min/μg/m^3^, 95% CI 0.08 to 0.67, *p*=0.02). The two-pollutant PM₂.₅ coefficient became null in both sexes (men: − 0.03 min/μg/m^3^, p=0.85; women: 0.05 min/μg/m^3^, p=0.73 (Supplementary Table S5).

We also validated the CAMS race-window estimates against ground-level monitoring data in five cities (Supplementary Material, Ground Monitor Validation). Across 68 matched race-years for NO₂ and 47 for PM₂.₅, CAMS showed moderate-to-strong rank-order agreement with monitors for NO₂ in all five cities (Pearson r = 0.52–0.82), while systematically underestimating absolute NO₂ concentrations relative to (frequently kerbside) monitoring stations. Agreement for PM₂.₅ was strong in Berlin, London and New York (r = 0.75–0.80) but weak in Boston (r = 0.09) and Chicago (r = 0.34). Because exposure enters our models through between-event contrasts, this rank-order agreement supports the use of CAMS as a valid index of relative race-day exposure; the weaker PM₂.₅ performance is relevant to the interpretation of the two-pollutant models (see Discussion).

### Heterogeneous Effects by Age and Performance Level

#### Age-Stratified Effects

**Fig. 3:**
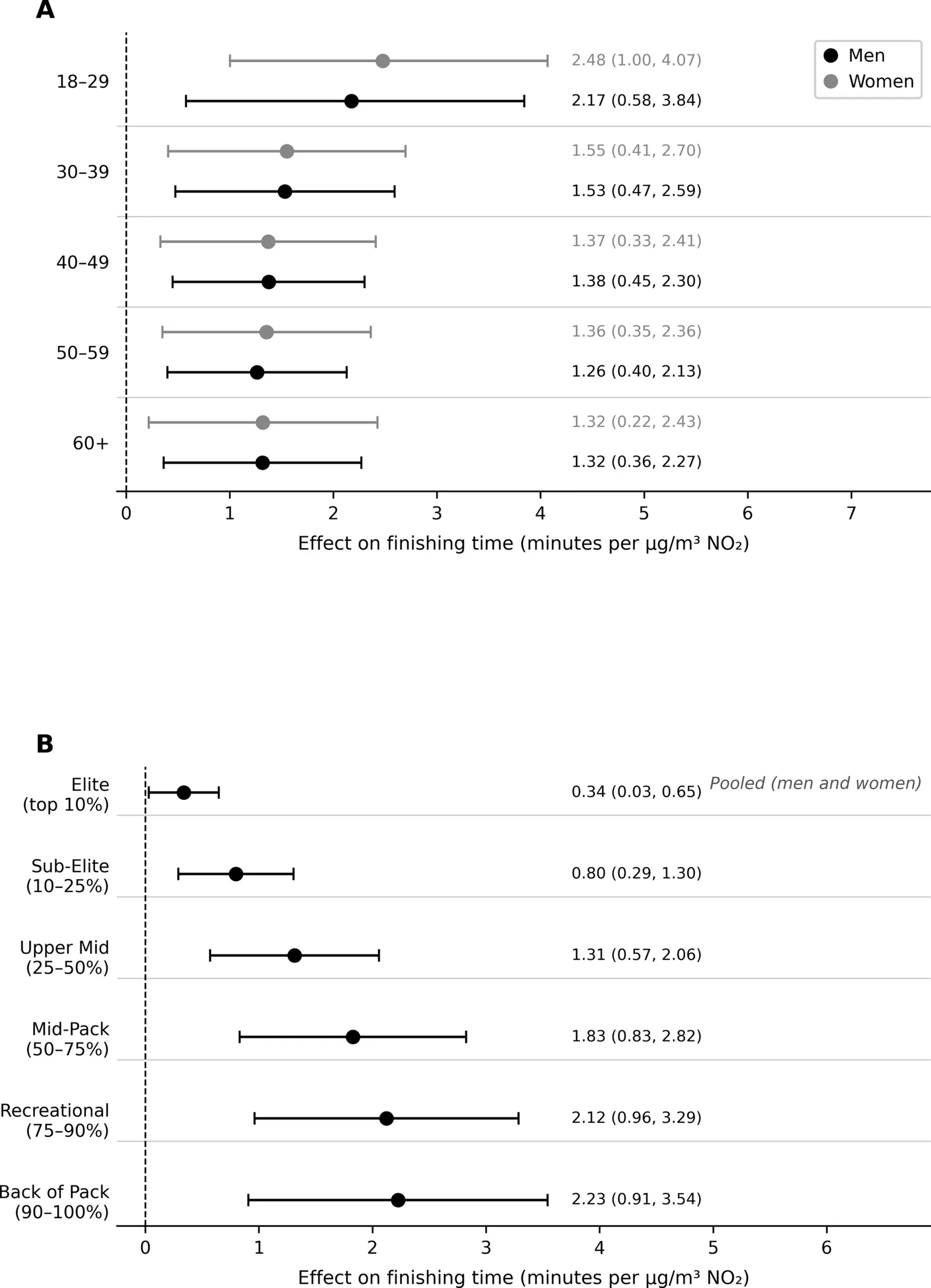
Forest plot of NO₂ effects on marathon finishing time by subgroup. **A** Effects by age group and **B** effects by performance level. Points show regression coefficients (minutes per µg/m^3^ NO₂) with 95% confidence intervals from linear mixed models. All models adjusted for heat index, wind speed, and year, with random intercepts for marathon–year events. Age-stratified estimates are shown for men

The association between NO₂ and finishing time attenuated with increasing age (Supplementary Table S3, Fig. 3A; estimates shown for men). Among men aged 18–29 years, each 1 µg/m^3^ increase in NO₂ was associated with 2.17 min slower finishing time (95% CI 0.58, 3.84), whereas among runners aged ≥60 years the corresponding estimate was 1.32 min (95% CI 0.36, 2.27). This gradient persisted when effects were expressed as a percentage of mean finishing time (0.87% at 18–29 years versus 0.45% at ≥60 years), indicating a genuinely larger proportional effect in younger runners rather than an artefact of differing baseline finishing times. PM₂.₅ associations were not significant across age groups.

#### Performance-Stratified Effects

Pollution–performance associations varied substantially by performance level (Supplementary Table S4, Fig. 3B). Elite runners (0–10th percentile) showed the smallest associations with NO₂ (0.34 min per µg/m^3^; 95% CI 0.03, 0.65; p=0.035), with effects increasing monotonically across performance strata to 2.12 min per µg/m^3^ (95% CI 0.91, 3.54) among back-of-pack runners (90–100th percentile). This gradient was also evident on the relative scale, rising from 0.18% of mean finishing time in elite runners to approximately 0.67% in recreational runners, confirming that the larger absolute effects in slower runners do not simply reflect their longer finishing times. PM₂.₅ associations were not statistically significant across performance strata.

### Distribution-Based Analyses

**Fig. 4:**
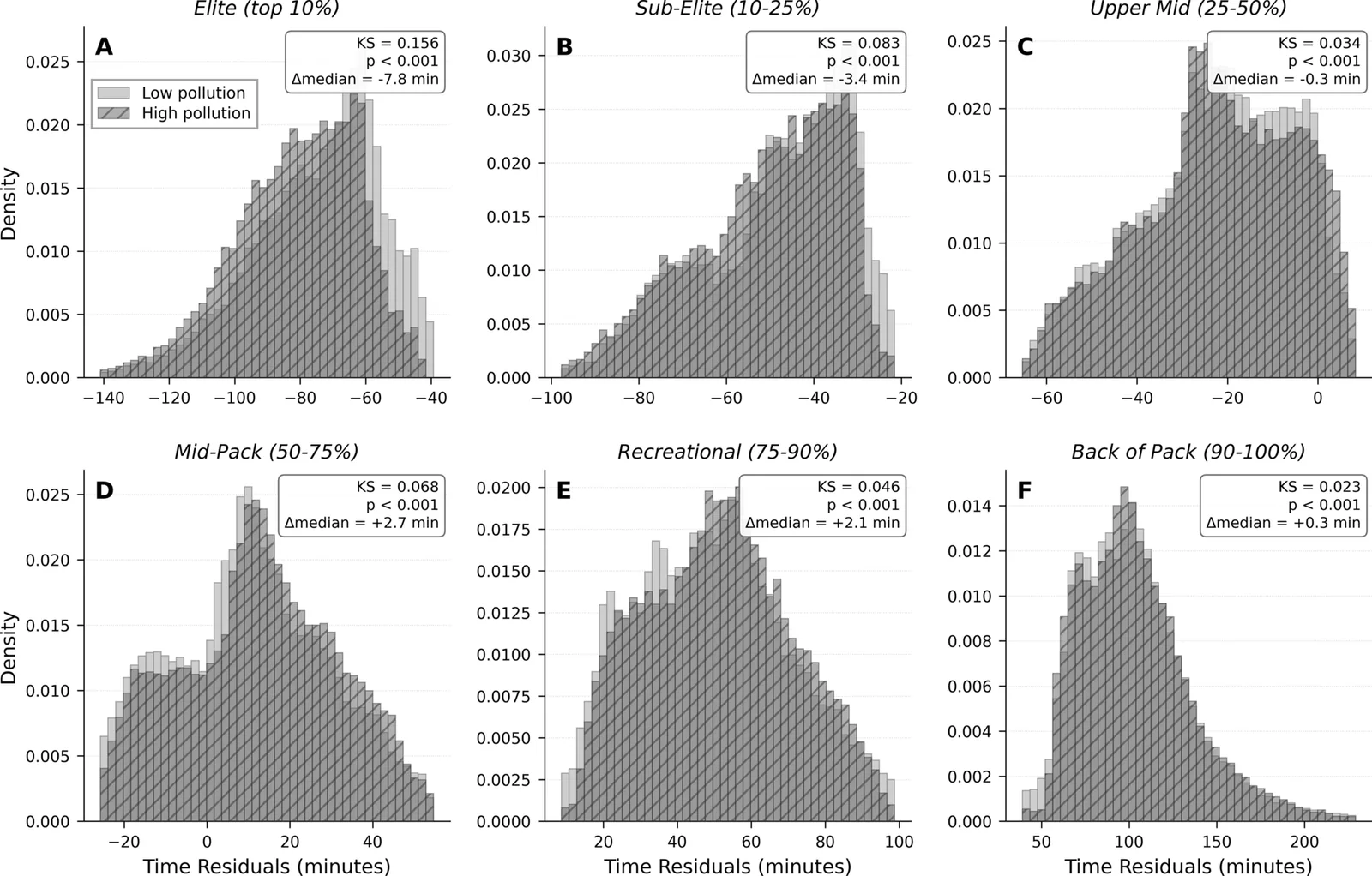
Cumulative distribution functions of weather-adjusted finishing times by NO₂ exposure level and performance category. High pollution defined as >75th percentile and low pollution as <25th percentile of NO₂ concentrations across all study events. Shaded areas show divergence between high- and low-pollution distributions. Kolmogorov–Smirnov (K–S) statistics and median time differences (minutes) are shown for each performance category

Weather-residualised Kolmogorov–Smirnov analysis demonstrated consistent rightward shifts in finishing time distribution under high NO₂ conditions (overall KS=0.055 (male) and 0.066 (female), both *p*<0.001; weather-residualised median difference: 9.5 min (male) and 10.1 min (female). Figure 4 illustrates these distributional shifts stratified by performance level. Distributional shifts were most pronounced among recreational runners and within individual marathon series that experienced greater year-to-year variation in NO₂ concentrations. Additional distributional results for PM₂.₅ are presented in Supplementary Table S2.

## Discussion

In this analysis of 2.76 million marathon performances across six World Marathon Majors, higher race-day ambient air pollution was associated with slower finishing times. Of the two pollutants examined, NO₂ showed the stronger and more confident association, with each 1 µg/m^3^ increase associated with a 1.4-to-1.6-min slower finishing time. PM₂.₅ associations were weaker and less consistent. These findings were most pronounced among recreational runners, and since pollution was assigned at the marathon–year event level, associations reflect between-event differences in environmental conditions.

### Comparison with Previous Marathon Research

Our findings extend prior large-scale analysis linking air pollution to endurance performance. El Helou et al. (2012) analysed 1,791,972 participants across six major marathons (Paris, London, Berlin, Boston, Chicago, New York) from 2001–2010 and identified NO₂ as an important environmental predictor alongside temperature [19]. Our study, spanning 2010–2024 and incorporating more than 2.7 million performances across Europe, US and Asia confirms NO₂ as a key pollutant associated with endurance performance outcomes.

With respect to particulate matter, Fleury et al. [9] recently analysed 2,564,811 performances from nine major US marathons (2003–2019), reporting that each 1 µg/m^3^ increase in race-day PM₂.₅ was associated with a 32-s slower finish time in men and 25-s slower finishing time in women, with effects more pronounced among faster-than-median finishers[9]. Our single-pollutant PM₂.₅ estimates were close to the null and not statistically significant (men: −8 s per µg/m^3^, 95% CI: −40 to 24 s), of broadly similar magnitude but not in a consistent direction. Notably, Fleury et al. did not examine NO₂, which our results identify as the stronger predictor. Earlier work by Marr and Ely[12], examining seven US marathons over 8–28 years, found that each 10 µg/m^3^ increase in PM₁₀ was associated with a 1.4% performance decrement (approximately 2–3 min for a 3-h marathon) in female runners, with no significant effects in males[12]. The sex difference was attributed to anatomical factors, where a women's smaller tracheas may facilitate greater particle deposition and irritation [12, 20].

An analysis of over 300,000 runners across 56 marathon events in China during 2014–2015, also found that during high-pollution conditions (often exceeding 100 µg/m^3^ PM₂.₅), average marathon runners required approximately 12 additional minutes to complete the race [10]. This substantially larger effect magnitude likely reflects the more severe pollution conditions in Chinese marathons compared to the other events in our sample (typically 5–30 µg/m^3^).

Cusick et al. (2023) demonstrated that training-period exposures to air pollution also influences performance, reporting a 12.8-s increase in 5,000-m race times among collegiate athletes associated with 21 days of elevated PM₂.₅ exposure (10.0 vs 5.0 µg/m^3^), as well as slower times with higher ozone exposure. These findings suggest that race-day pollution may represent only part of the total environmental burden affecting endurance performance, and that our estimates likely reflect acute rather than cumulative exposure across training cycles.

### Heterogeneity Across Performance Levels

Associations between NO_2_ and finishing time varied substantially by performance level. Recreational runners demonstrated substantially larger NO₂ associations (2.12 min per µg/m^3^) than elite runners (0.34 min per µg/m^3^), with effects increasing progressively across performance strata. This pattern differs from Fleury et al. (2025), who reported stronger PM₂.₅ effects among faster-than-median finishers using broader categorisation. Our finer stratification suggests that vulnerability may vary across the full performance spectrum and differ by pollutant type.

Several mechanisms may explain greater susceptibility among recreational runners. First, slower runners remain on course longer, potentially accumulating greater pollutant dose during race day [20]. Second, elite athletes typically possess higher cardiopulmonary efficiency and greater physiological reserve, which may mitigate acute environmental stressors. Third, sustained high intensity training is associated with enhanced antioxidant capacity, improved pulmonary clearance, and adaptive immune responses, which could confer partial protection against pollution-related oxidative and inflammatory effects [21, 22]. In contrast, recreational runners may have lower training volumes and physiological reserve, potentially amplifying performance impacts during prolonged exposure. Furthermore, recreational runners are more likely to train in the same urban environments where marathons are held, accumulating chronic pollution exposure during training, whereas elite runners typically train in cleaner environments and compete internationally [8].

From a broader perspective, these findings have public health relevance. Recreational runners more closely resemble the general population engaging in outdoor physical activity.

### Age-Related Heterogeneity

Interestingly, we found younger runners (18–29 years) showed larger NO₂ effects (2.17 min per µg/m^3^) than older runners (≥ 60 years: 1.32 min per µg/m^3^). This appears paradoxical given extensive literature demonstrating that older adults (≥ 65 years) exhibit greater susceptibility to pollution-induced cardiovascular events, attributed to age-related declines in physiological reserve, arterial elasticity, and compensatory capacity, alongside higher prevalence of cardiovascular comorbidities [23, 24]. One possible explanation for the attenuated associations observed among older runners is healthy selection effect within marathon populations. Individuals aged ≥60 years who complete marathons likely represent a highly selected subset of their age cohort, those who have maintained exceptional cardiovascular fitness, avoided chronic disease, and possessed genetic or lifestyle factors conferring protection against age-related decline. Such individuals may possess greater physiological reserve relative to age-matched peers potentially reducing susceptibility to acute environmental stressors during endurance exercise. In contrast, the youngest age group (18–29 years) includes a broader spectrum of training status and physiological resilience, including recreational participants undertaking early career marathons. Greater heterogeneity in fitness and adaptation within this group may partly account for the larger observed associations.

This interpretation is consistent with evidence suggesting that long-term endurance training may enhance antioxidant capacity, improve pulmonary clearance, and modulate inflammatory and oxidative stress pathways, potentially mitigating some acute effects of air pollution exposure [21, 22]. However, these mechanisms remain speculative in the context of the present study.

Notably, race-day NO₂ concentrations in our sample were relatively low (median 7.3 µg/m^3^, 25th to 75th percentile 5.2–10.3 µg/m^3^) compared with typical urban annual mean concentrations, likely reflecting that marathons are predominantly held on weekend mornings when traffic volumes are reduced, and race routes are often closed to vehicular traffic. As discussed below, the detection of measurable performance associations even at these comparatively modest concentrations is itself noteworthy.

### Pollutant Sources, Ozone, and the Two-Pollutant Models

NO₂ at these events is overwhelmingly traffic-derived, generated by combustion in the dense road networks surrounding urban marathon courses, whereas race-day PM₂.₅ represents a more heterogeneous mixture of traffic emissions, residential and commercial combustion, and regional secondary aerosol, with seasonal contributions from springtime dust transport and biomass sources. This compositional difference may partly explain why all of the spring-held majors in our sample exhibited higher peak PM₂.₅ concentrations, and most exhibited higher NO₂, than the autumn events. It is also worth noting that NO₂ and sunlight drive the photochemical formation of ground-level ozone (O₃), itself a respiratory irritant associated with impaired exercise performance; O₃ was not available in our exposure data and represents a plausible co-exposure that future work should incorporate.

To place these concentrations in context, race-day NO₂ and PM₂.₅ levels in our sample were generally low to moderate relative to the 2021 World Health Organization air quality guideline levels [25] (24-h means of 25 µg/m^3^ for NO₂ and 15 µg/m^3^ for PM₂.₅), with most events falling near or below these thresholds. Although such short-window guideline values are not directly applicable to individuals during exercise, the detection of measurable performance associations at these comparatively modest concentrations suggests that effects could be greater under the higher-exposure conditions encountered during routine urban training or in cities with poorer air quality. However, given the potential uncertainty associated with the relatively low spatial resolution of the exposure data, and the likelihood that pollutant concentrations vary considerably within individual grid cells, care should be taken when extrapolating these findings to other exposure settings or concentration ranges.

In two-pollutant models, the PM₂.₅ coefficient reversed sign and became statistically significant in the negative direction. We do not interpret this as evidence that PM₂.₅ improves performance. Several considerations argue against causal reading. The single-pollutant PM₂.₅ association is null, and NO₂ and PM₂.₅ are only modestly correlated (r = 0.32), making collinearity an unlikely explanation. When two error-prone, positively correlated exposures that share an emission source are entered into the same model, the more accurately measured pollutant can absorb the shared signal and induce a spurious negative coefficient for the more poorly measured one [26, 27]; consistent with this, our ground-monitor validation showed materially weaker measurement performance for PM₂.₅ than for NO₂ in two of five cities (Boston r = 0.09, Chicago r = 0.34) and our new sensitivity excluding all Boston events confirms that the negative two-pollutant PM₂.₅ coefficient disappears when Boston’s high-error events are removed. Negative confounding by season and meteorology may further contribute. We therefore regard the single pollutant estimates as the primary results.

### Biological Mechanisms

The mechanisms linking air pollution to impaired endurance performance likely involve multiple interconnected pathways including systemic inflammation and oxidative stress, cardiovascular endothelial dysfunction and impaired respiratory function such as reduced gas exchange efficiency or increases in airway resistance [5, 28]. A detailed discussion of biological mechanisms is provided in Supplementary File S2. Given plausible mechanistic pathways and consistency across studies of marathons and endurance running, we consider our findings most likely represent a causal association.

### Clinical Implications

These findings have practical implications for multiple stakeholders. Race organisers should consider incorporating air quality forecasts alongside temperature and humidity when planning event schedules, and urban traffic management policies are particularly relevant given that NO₂ is primarily a traffic-related pollutant, with potential for real-time pollution advisories during races. Adjusting race start times to earlier hours, when traffic-related pollution concentrations are typically lower, represents another practical mitigation strategy. Athletes and coaches, particularly those working with recreational runners, may benefit from adjusting pacing strategies or training locations on high-pollution days. From a public health perspective, as recreational runners most closely resemble the general exercising population, air quality represents an important and currently under-studied environmental consideration [29] for outdoor physical activity in urban settings. Limiting traffic in the wider vicinity of the marathon if feasible, would contribute to better running conditions.

### Strengths and Limitations

This study benefits from its scale, multinational scope, and inclusion of 2.76 million performances across three continents over 15 years, combined with complementary distributional analyses enabling detailed evaluation of heterogeneity. However, several limitations warrant consideration. Pollution exposure was assigned at the individual event level to geographical coordinates at the start of the race using relatively coarse spatial resolution data; and individual-level spatial variability along race courses and temporal variability across finishing times were not captured. The CAMS reanalysis spatial resolution (approximately 11 km) is coarse relative to a 42-km marathon route, which may be particularly important for NO₂ given its greater spatial variability at small geographical scales compared to PM₂.₅. This may have introduced exposure misclassification, especially for NO_2_ with generally higher variability than PM_2.5_ and may have led to under-estimation of air pollution effects. Exposure assessment was limited to acute race-day conditions and did not account for cumulative training-period exposure. The attenuated associations observed in older runners may reflect selection effects within marathon populations, potentially limiting generalisability. Because the publicly available results do not contain persistent individual identifiers across events, we were unable to identify runners who competed in more than one marathon or year, and our models therefore treat all performances as independent conditional on the event-level random intercept. Repeated participation by the same individuals would introduce a degree of within-person correlation not captured by this structure; this is expected to affect the precision of estimates (potentially rendering standard errors mildly anti-conservative) rather than to bias the point estimates, since pollution exposure is assigned at the event level and identified from between-event variation. Finally, although models adjusted for key meteorological variables, residual confounding by unmeasured environmental or contextual factors (including co-pollutants such as ozone) cannot be excluded.

## Conclusion

In this large, multinational analysis, higher race-day ambient air pollution, particularly NO₂, was associated with slower marathon finishing times, with strongest effects among recreational runners. As participation in mass endurance events expands globally, consideration of air quality alongside temperature and humidity is relevant for race planning, athlete guidance, and public health policies promoting awareness of environmental factors during outdoor physical activity.

## Supplementary Information


Supplementary Material 1.

## Data Availability

The marathon performance data were obtained from publicly available official results websites. Air pollution data are available from the Copernicus Atmosphere Monitoring Service (https://www.copernicus.eu/). Weather data are available from the Open-Meteo Historical Weather API (https://open-meteo.com/). The complete analytical code is available in Supplementary File S1. Processed datasets are available from the corresponding author upon reasonable request.

## Abbreviations

API: Application Programming Interface
CAMS: Copernicus Atmosphere Monitoring Service
CI: Confidence interval
COVID-19: Coronavirus disease 2019
ERA5: ECMWF Reanalysis v5
IQR: Interquartile range
K-S: Kolmogorov–Smirnov
LMM: Linear mixed-effects model
NO₂: Nitrogen dioxide
NOAA: National Oceanic and Atmospheric Administration
PM₂.₅: Fine particulate matter (≤ 2.5 µm aerodynamic diameter)
PM₁₀: Particulate matter (≤ 10 µm aerodynamic diameter)
Q–Q: Quantile–quantile

## Code Availability

The complete Python and R codebase used for data collection, analysis, and visualisation is available in the Supplementary Materials (Supplementary File S1) and from the corresponding author.
