# Supplementary material for: Air Pollution Impairs Marathon Performance: A Cross-Sectional Analysis of 2.7 Million Finishers Across Six World Marathon Majors
Source: Sports Med Open. 2026 Aug 29;12:128. doi: 10.1186/s40798-026-01099-6 (PMC13525978; doi:10.1186/s40798-026-01099-6)
Supplement: Supplementary file 1 [file 40798_2026_1099_MOESM1_ESM.pdf]

Supplementary Material

Air Pollution Impairs Marathon Performance: A Cross-Sectional Analysis of 2.7 Million Finishers Across Six World Marathon Majors

James A. Donaldson1-3,8, Samuel Cai[2–5], Joshua Vande Hey[2–5], Anna Hansell[2–5], Thomas Yates[6,7], Andre Ng[1,3,4], Jamie O'Driscoll[6,7]

Sports Medicine - Open — Manuscript SMOA-D-26-00424

Affiliations:

1. Department of Cardiovascular Sciences, University of Leicester, Leicester, United Kingdom
2. Centre for Environmental Health and Sustainability, Division of Public Health and Epidemiology, School of Medical Sciences, University of Leicester, Leicester, United Kingdom
3. NIHR Leicester Biomedical Research Centre, Leicester, United Kingdom
4. Leicester British Heart Foundation Centre of Research Excellence, University of Leicester, Glenfield Hospital, Leicester, United Kingdom
5. NIHR Health Protection Research Unit in Chemical Threats and Hazards, University of Leicester, United Kingdom
6. Diabetes Research Centre, College of Life Sciences, University of Leicester, Leicester, United Kingdom
7. Leicester Diabetes Centre, Leicester General Hospital, University Hospitals of Leicester NHS Trust, Leicester, United Kingdom

Corresponding author: James A. Donaldson (jad72@leicester.ac.uk)

Contents

- Supplementary File S1 — Code Availability
- Supplementary File S2 — Extended Results (biological mechanisms and additional discussion)
- Supplementary Table S1 — Within-marathon Kolmogorov–Smirnov tests (NO_2)
- Supplementary Table S2 — Kolmogorov–Smirnov tests ($PM_{2.5}$)
- Supplementary Table S3 — NO_2 effects by age
- Supplementary Table S4 — NO_2 effects by performance level
- Supplementary Table S5 — Sensitivity analyses (including exclusion of Boston 2013)
- Supplementary Table S6 — Marathon comparison
- Supplementary Table S7 — Timing basis by marathon series
- Supplementary Table S8 — NO_2 effects as a percentage of mean finishing time
- Supplementary Table S9 — CAMS vs ground-monitor agreement, and Ground Monitor Validation section

Code for the project is publicly available to view at https://github.com/jamesad90/marathon-pollution

Air Pollution and Marathon Performance

Code repository for the research study by Donaldson et al. examining the association between ambient air pollution (NO2 and PM2.5) and marathon finish times across the six World Marathon Majors (2010–2024).

This repository is provided in accordance with the BMJ's data sharing and code availability policy.

Study Overview

- **Design:** Retrospective observational study
- **Exposure:** Race-day NO2 and PM2.5 concentrations (Copernicus CAMS Global Reanalysis EAC4)
- **Outcome:** Individual marathon finish time
- **Marathons:** Boston, London, Berlin, Chicago, New York City, Tokyo
- **Period:** 2010–2024
- **Statistical approach:** Linear mixed models with random intercepts per marathon-year event, adjusted for heat index (natural spline), wind speed, and secular trends; linear quantile mixed models at multiple performance percentiles; subgroup analyses by sex, age, and performance level

Repository Structure

```
marathon-pollution/
├── api_scraper.py # Runner results collection (runzy.com API)
├── marathon_pollution_data.py # Pollution data download (Copernicus CAMS/CDS API)
├── get_marathon_weather_openmeteo.py # Weather data collection (Open-Meteo API)
├── merge_comprehensive_data.py # Merges runner, pollution, and weather data
├── mixed_models_analysis.R # Primary LMM and LQMM analyses
├── age_ability_mixed_models.R # Subgroup analyses by age and performance level
├── sensitivity_analyses.R # Sensitivity and robustness checks
├── two_pollutant_models.R # Two-pollutant models and pollutant correlations
├── random_intercept_tests.R # Likelihood ratio tests and marathon-specific models
├── pollution_nc_files/ # Raw CAMS netCDF files
└── marathon gpx files/ # GPX route files for each marathon
```

Data Pipeline

Scripts should be run in the following order:

1. **api_scraper.py** — Scrapes individual runner finish times (name, time, sex, age, place) for all marathons and years from the runzy.com/marathonguide.com API.
2. **marathon_pollution_data.py** — Downloads race-day PM2.5, PM10, and NO2 concentrations from Copernicus CAMS EAC4 at four time points per day across a bounding box around each marathon city. Requires a Copernicus CDS API key (see below).
3. **get_marathon_weather_openmeteo.py** — Retrieves hourly temperature, humidity, wind speed, wind direction, precipitation, and surface pressure from the Open-Meteo historical archive, averaged over race hours (06:00–16:00).

4. **merge_comprehensive_data.py** — Joins runner results with pollution and weather data by marathon and year. Converts finish times to minutes and produces the final analysis-ready dataset.

Statistical Analyses

All R scripts read from the merged dataset and write results to a results/ directory.

Script	Description
mixed_models_analysis.R	Primary sex-stratified LMMs and LQMMs (1st–90th percentile) for NO2 and PM2.5
age_ability_mixed_models.R	Stratified analyses by age group (18–29 to 60+) and performance level (elite to back-of-pack)
sensitivity_analyses.R	Alternative heat index specifications, exclusion of extreme pollution events, alternative exposure windows
two_pollutant_models.R	Simultaneous two-pollutant models; NO2–PM2.5 correlations
random_intercept_tests.R	Likelihood ratio tests for random effects; ICC; marathon-specific models

Requirements

Python

- Python 3
- pandas, numpy, requests, xarray, netCDF4, cdsapi

R

- data.table, lme4, lmerTest, lqmm, splines, ggplot2, dplyr, parallel

Copernicus CDS API

To reproduce the pollution data download, a Copernicus Climate Data Store account and API key are required. Configure credentials in ~/.cdsapirc as described at https://cds.climate.copernicus.eu/api-how-to.

Data Availability

Raw runner results were obtained from publicly available marathon results databases. Pollution data are from the Copernicus Atmosphere Monitoring Service (CAMS) Global Reanalysis (EAC4). Weather data are from the Open-Meteo historical weather API.

License

This code is made available for the purposes of transparency and reproducibility of the associated research study.

Supplementary File S2: Extended Results and Biological Mechanisms

S2.1 Biological Mechanisms

The mechanisms linking air pollution to impaired endurance performance likely involve multiple interconnected pathways:

Systemic inflammation and oxidative stress: Inhalation of NO_2 and $PM_{2.5}$ triggers pulmonary inflammation that rapidly becomes systemic, with elevated circulating levels of C-reactive protein, interleukin-6, and tumour necrosis factor-α detectable within hours of exposure. These inflammatory mediators promote oxidative stress through increased reactive oxygen species production, depleting antioxidant reserves that are already under strain during prolonged exercise.

Cardiovascular and endothelial dysfunction: NO_2 exposure directly impairs endothelial function through oxidative inactivation of nitric oxide, a critical vasodilator. Münzel et al. (2018) demonstrated that gaseous pollutants reduce flow-mediated dilation and increase arterial stiffness. During marathon running, when cardiac output must increase 4–5 fold, impaired vasodilation could meaningfully reduce muscle perfusion and oxygen delivery. Karoui et al. (2020) showed that NO_2 inhalation induces cardiac mitochondrial reactive oxygen species production and promotes coronary endothelial dysfunction.

Respiratory impairment: Particulate matter deposited in the airways increases resistance and may reduce gas exchange efficiency. During exercise, the shift to oral breathing bypasses nasal filtration, allowing greater pollutant penetration to the lower airways (Saibene et al., 1978). Cruz et al. (2021) provided evidence that exercise substantially increases the deposition of fine particles in the pulmonary region.

Supplementary Table S1: Within-Marathon Kolmogorov-Smirnov Tests for NO_2 Exposure by Sex

Marathon	Sex	KS Statistic	p-value	n (High NO_2)	n (Low NO_2)	Median NO_2 Split (μg/m³)
Berlin	F	0.0382	<0.001	62,733	80,922	6.12
Berlin	M	0.0362	<0.001	170,786	203,100	6.12
Boston	F	0.0329	<0.001	73,576	70,100	6.56
Boston	M	0.0229	<0.001	95,494	88,226	6.56
Chicago	F	0.0775	<0.001	101,820	135,985	7.60
Chicago	M	0.1018	<0.001	119,418	159,772	7.60
London	F	0.0529	<0.001	96,568	112,949	10.89
London	M	0.0570	<0.001	158,378	170,415	10.89
Nyc	F	0.0087	<0.001	106,487	147,023	7.33
Nyc	M	0.0124	<0.001	155,177	205,981	7.33
Tokyo	F	0.0320	<0.001	23,041	32,198	7.16
Tokyo	M	0.0178	<0.001	79,714	106,690	7.16

Note: KS = Kolmogorov-Smirnov. Results based on Method 2 (3-hour race-window NO_2 exposure). High/Low NO_2 groups were split at the within-marathon median NO_2 concentration. All p-values are two-sided. M = Male; F = Female.

Supplementary Table S2: Weather-Residualised Kolmogorov-Smirnov Tests for PM$_2$.5 Exposure

Marathon	Sex	N (High PM$_2$.5)	N (Low PM$_2$.5)	KS Statistic	p-value	Median Diff (s)
**All**	**M**	**423,219**	**485,249**	**0.0374**	**<0.001**	**+94.4**
**All**	**F**	**211,285**	**332,566**	**0.0195**	**<0.001**	**+72.9**
Berlin	M	26,604	90,595	0.0465	<0.001	+90.1
Berlin	F	7,397	36,190	0.0532	<0.001	+89.9
Boston	M	69,547	13,251	0.0324	<0.001	+19.2
Boston	F	54,904	9,956	0.0141	0.071	+37.0
Chicago	M	23,934	150,414	0.0084	0.106	-38.0
Chicago	F	20,650	129,475	0.0127	0.007	-57.3
London	M	89,383	22,763	0.0252	<0.001	+91.8
London	F	51,139	16,215	0.0243	<0.001	+51.3
NYC	M	27,347	208,226	0.0438	<0.001	-31.1
NYC	F	21,956	140,730	0.0381	<0.001	-12.5
Tokyo	M	186,404	0	—	—	—
Tokyo	F	55,239	0	—	—	—

Note: $PM_{2.5}$ high/low groups defined using >75th and <25th percentiles of race-day $PM_{2.5}$ concentration across all events. Weather residuals obtained by regressing finishing time on heat index (natural spline, 5 df) and wind speed within each marathon-year event. KS = Kolmogorov-Smirnov. — = not estimable (Tokyo had no low-$PM_{2.5}$ events). Median Diff = median residual of high $PM_{2.5}$ group minus median residual of low $PM_{2.5}$ group (seconds).

Supplementary Table S3: NO_2 Effects on Marathon Finishing Time by Age Group

Age Group	Sex	N	Coefficient (s/ μg/m³)	95% CI (s)	Coefficient (min/μg/m³)	95% CI (min)	IQR Effect (min)
18-29	F	115,482	148.66	(60.22, 244.13)	2.4776	(1.0037, 4.0689)	12.63
18-29	M	101,753	130.45	(34.74, 230.53)	2.1742	(0.5790, 3.8422)	11.09
30-39	F	209,673	93.11	(24.38, 161.84)	1.5518	(0.4063, 2.6974)	7.91
30-39	M	325,658	91.99	(28.46, 155.50)	1.5331	(0.4744, 2.5917)	7.82
40-49	F	199,902	82.27	(19.95, 144.61)	1.3712	(0.3325, 2.4101)	6.99
40-49	M	392,109	82.56	(26.97, 138.15)	1.3760	(0.4495, 2.3025)	7.02
50-59	F	103,662	81.35	(21.03, 141.67)	1.3558	(0.3505, 2.3612)	6.91
50-59	M	255,038	75.86	(23.96, 127.75)	1.2643	(0.3993, 2.1292)	6.45
60+	F	24,418	79.12	(13.18, 145.59)	1.3187	(0.2196, 2.4266)	6.72
60+	M	87,139	79.04	(21.84, 136.24)	1.3173	(0.3639, 2.2706)	6.72

Note: Results from linear mixed models with marathon-year as random intercept. Method 2 (3-hour race-window) NO_2 exposure. IQR effect calculated using NO_2 IQR = 5.10 μg/m³. Coefficients represent change in finishing time per 1 μg/m³ increase in NO_2. 95% CI = 95% confidence interval. M = Male; F = Female.

Supplementary Table S4: NO_2 Effects on Marathon Finishing Time by Performance Level

Performance Group	N	Coefficient (s/μg/m³)	95% CI (s)	Coefficient (min/μg/m³)	95% CI (min)	IQR Effect (min)	p-value
Elite (top 10%)	275,636	20.37	(1.86, 38.88)	0.3395	(0.0310, 0.6481)	1.73	0.035
Sub-Elite (10-25%)	413,658	47.85	(17.43, 78.27)	0.7975	(0.2906, 1.3045)	4.07	0.003
Upper Mid (25-50%)	689,353	78.90	(34.30, 123.50)	1.3150	(0.5716, 2.0584)	6.71	0.001
Mid-Pack (50-75%)	689,292	109.65	(49.87, 169.44)	1.8276	(0.8311, 2.8240)	9.32	<0.001
Recreational (75-90%)	413,608	127.44	(57.72, 197.16)	2.1241	(0.9621, 3.2861)	10.83	<0.001
Back of Pack (90-100%)	275,801	133.52	(54.48, 212.55)	2.2253	(0.9080, 3.5426)	11.35	0.002

Note: Results from linear mixed models with marathon-year as random intercept. Method 2 (3-hour race-window) NO_2 exposure. Performance groups defined by within-race finishing time percentiles (pooled across sexes). IQR effect calculated using NO_2 IQR = 5.10 μg/m³. Coefficients represent change in finishing time per 1 μg/m³ increase in NO_2. 95% CI = 95% confidence interval.

Supplementary Table S5: Sensitivity Analyses for NO_2 and $PM_{2.5}$ Effects on Marathon Finishing Time

Specification	Pollutant	Sex	Coefficient (s/μg/m³)	95% CI	N
Main model (ns df=5)	NO2	M	86.04	(39.55, 132.53)	1,713,151
	NO2	F	93.39	(37.82, 148.97)	1,043,402
	PM2.5	M	-8.04	(-40.08, 24.00)	1,713,151
	PM2.5	F	-8.19	(-45.78, 29.41)	1,043,402
Quadratic heat index	NO2	M	84.83	(40.38, 129.29)	1,713,151
	NO2	F	90.51	(37.34, 143.67)	1,043,402
	PM2.5	M	-7.16	(-38.14, 23.81)	1,713,151
	PM2.5	F	-7.52	(-43.83, 28.78)	1,043,402
Spline df=3	NO2	M	84.90	(40.13, 129.66)	1,713,151
	NO2	F	90.36	(36.82, 143.90)	1,043,402
	PM2.5	M	-7.29	(-38.65, 24.08)	1,713,151
	PM2.5	F	-7.84	(-44.60, 28.91)	1,043,402
Spline df=4	NO2	M	85.43	(40.09, 130.77)	1,713,151
	NO2	F	91.27	(37.04, 145.50)	1,043,402
	PM2.5	M	-7.20	(-38.84, 24.43)	1,713,151
	PM2.5	F	-7.77	(-44.84, 29.30)	1,043,402
Spline df=6	NO2	M	84.69	(37.66, 131.72)	1,713,151
	NO2	F	93.34	(37.27, 149.41)	1,043,402

	PM2.5	M	-7.99	(-40.24, 24.27)	1,713,151
	PM2.5	F	-8.25	(-46.33, 29.82)	1,043,402
Spline df=7	NO2	M	84.00	(36.38, 131.61)	1,713,151
	NO2	F	92.49	(35.75, 149.22)	1,043,402
	PM2.5	M	-6.76	(-39.65, 26.13)	1,713,151
	PM2.5	F	-5.97	(-44.63, 32.69)	1,043,402
Linear heat index	NO2	M	86.99	(42.97, 131.01)	1,713,151
	NO2	F	91.41	(39.03, 143.79)	1,043,402
	PM2.5	M	-9.34	(-40.09, 21.41)	1,713,151
	PM2.5	F	-8.88	(-44.67, 26.91)	1,043,402
Excl. extreme pollution (5-95th)	NO2	M	82.59	(18.27, 146.91)	1,572,630
	NO2	F	95.77	(19.87, 171.66)	953,144
	PM2.5	M	-26.85	(-71.58, 17.89)	1,551,819
	PM2.5	F	-33.71	(-86.74, 19.31)	945,486
Excl. berlin	NO2	M	87.68	(30.37, 144.98)	1,339,265
	NO2	F	93.78	(24.60, 162.95)	899,747
	PM2.5	M	-6.56	(-44.79, 31.66)	1,339,265
	PM2.5	F	-7.11	(-51.80, 37.58)	899,747
Excl. boston	NO2	M	60.47	(37.20, 83.75)	1,529,431
	NO2	F	66.33	(42.55, 90.12)	899,726
	PM2.5	M	17.46	(0.26, 34.65)	1,529,431

	PM2.5	F	22.70	(5.09, 40.31)	899,726
Excl. chicago	NO2	M	91.57	(36.03, 147.10)	1,433,961
	NO2	F	97.96	(31.15, 164.77)	805,597
	PM2.5	M	1.44	(-39.76, 42.63)	1,433,961
	PM2.5	F	2.99	(-45.40, 51.38)	805,597
Excl. london	NO2	M	71.10	(9.84, 132.37)	1,384,358
	NO2	F	67.79	(-3.45, 139.02)	833,885
	PM2.5	M	-49.79	(-91.93, -7.65)	1,384,358
	PM2.5	F	-61.83	(-109.21, -14.45)	833,885
Excl. nyc	NO2	M	130.34	(66.84, 193.83)	1,351,993
	NO2	F	160.15	(81.95, 238.35)	789,892
	PM2.5	M	3.03	(-37.16, 43.22)	1,351,993
	PM2.5	F	3.33	(-46.12, 52.78)	789,892
Excl. tokyo	NO2	M	70.86	(18.84, 122.88)	1,526,747
	NO2	F	81.28	(17.39, 145.18)	988,163
	PM2.5	M	-28.93	(-61.82, 3.96)	1,526,747
	PM2.5	F	-29.39	(-69.81, 11.03)	988,163
Excl. Boston 2013	NO_2	M	88.27	(44.68, 131.87)	1,703,030
	NO_2	F	95.96	(44.01, 147.91)	1,036,743
	$PM_{2.5}$	M	-1.55	(-32.75, 29.64)	1,703,030
	$PM_{2.5}$	F	-0.24	(-36.62, 36.15)	1,036,743
	NO_2	M	111.63		1,703,030

Excl. Boston 2013 (two-pollutant)				(64.54, 158.71)	
	NO_2	F	120.04	(63.30, 176.78)	1,036,743
	$PM_{2.5}$	M	-32.86	(-62.45, -3.28)	1,703,030
	$PM_{2.5}$	F	-33.84	(-69.42, 1.75)	1,036,743
Excl. all Boston events	NO_2	M	60.47	(37.20, 83.75)	1,529,431
	NO_2	F	66.33	(42.55, 90.12)	899,726
	$PM_{2.5}$	M	17.46	(0.26, 34.65)	1,529,431
	$PM_{2.5}$	F	22.70	(5.09, 40.31)	899,726
Excl. all Boston events (two-pollutant)	NO_2	M	61.87	(34.44, 89.30)	1,529,431
	NO_2	F	63.78	(35.78, 91.77)	899,726
	$PM_{2.5}$	M	-1.65	(-18.19, 14.90)	1,529,431
	$PM_{2.5}$	F	3.01	(-13.87, 19.90)	899,726

Note: All models use linear mixed models with marathon-year as random intercept. Method 2 (3-hour race-window) pollution exposure. Main model uses natural spline with df=5 for heat index. Spline df=3/4/6/7 = alternative heat index spline degrees of freedom. Excl. extreme pollution excludes observations outside 5th–95th percentile of pollution distribution. Leave-one-out analyses exclude one marathon city at a time. Excl. Boston 2013 excludes the 2013 Boston Marathon (halted before all participants finished); Excl. all Boston events excludes all 14 Boston Marathon editions (2010–2024) to address potentially weaker CAMS $PM_{2.5}$ agreement at Boston (ground-monitor Pearson $r = 0.09$); two-pollutant rows include NO_2 and $PM_{2.5}$ simultaneously. NO_2 = nitrogen dioxide; $PM_{2.5}$ = particulate matter ≤2.5 μm. M = Male; F = Female. 95% CI = 95% confidence interval (estimate ± 1.96 × standard error).

Supplementary Table S6: Marathon-Specific NO_2 and $PM_{2.5}$ Exposure Statistics (Method 2)

Marathon	Years (N events)	NO_2 Mean (μg/m³)	NO_2 SD	NO_2 Min	NO_2 Max	$PM_{2.5}$ Mean (μg/m³)	$PM_{2.5}$ SD	$PM_{2.5}$ Min	$PM_{2.5}$ Max
Berlin	2010-2024 (N=14)	6.38	2.17	3.28	11.80	10.41	4.40	3.16	18.11
Boston	2010-2024 (N=14)	7.03	3.10	2.84	12.95	15.53	9.20	4.37	37.02
Chicago	2010-2024 (N=14)	10.47	8.60	3.86	31.98	7.33	7.02	0.00	22.69
London	2010-2024 (N=15)	12.04	6.30	3.55	25.94	15.76	12.16	4.64	50.34
Nyc	2010-2024 (N=13)	11.14	8.91	3.42	32.29	5.41	4.78	0.02	17.40
Tokyo	2010-2024 (N=14)	9.23	5.71	1.97	18.84	25.13	9.84	8.88	43.75

Individual Event Pollution Values

Marathon	Year	NO_2 (μg/m³)	$PM_{2.5}$ (μg/m³)
Berlin	2010	11.80	18.11
Berlin	2011	8.40	14.44
Berlin	2012	4.94	9.18
Berlin	2013	4.08	3.16
Berlin	2014	7.95	9.81
Berlin	2015	5.41	4.64
Berlin	2016	6.09	10.05
Berlin	2017	6.58	14.95
Berlin	2018	6.16	11.63
Berlin	2019	5.34	9.65
Berlin	2021	7.02	13.99
Berlin	2022	7.75	13.44
Berlin	2023	4.49	8.58
Berlin	2024	3.28	4.03
Boston	2010	4.67	10.44
Boston	2011	5.80	18.73

Boston	2012	12.95	6.02
Boston	2013	10.32	16.33
Boston	2014	7.32	11.83
Boston	2015	6.20	28.38
Boston	2016	10.11	9.53
Boston	2017	4.75	16.51
Boston	2018	3.03	37.02
Boston	2019	6.91	21.23
Boston	2021	4.46	7.48
Boston	2022	2.84	4.37
Boston	2023	8.04	8.75
Boston	2024	10.99	20.76
Chicago	2010	27.08	15.36
Chicago	2011	31.98	14.08
Chicago	2012	4.25	6.39
Chicago	2013	7.77	0.49
Chicago	2014	6.35	0.28
Chicago	2015	7.94	0.00
Chicago	2016	7.60	4.67
Chicago	2017	15.07	0.00
Chicago	2018	8.91	22.69
Chicago	2019	3.86	2.86
Chicago	2021	8.77	13.38
Chicago	2022	7.37	3.29
Chicago	2023	4.34	9.42
Chicago	2024	5.22	9.70
London	2010	25.94	30.26
London	2011	24.85	50.34
London	2012	9.82	11.63
London	2013	15.38	21.81
London	2014	10.50	14.82

London	2015	12.04	9.89
London	2016	7.50	13.40
London	2017	12.87	14.93
London	2018	14.99	25.95
London	2019	5.06	7.87
London	2020	9.18	4.64
London	2021	9.16	6.82
London	2022	11.28	4.99
London	2023	8.49	11.02
London	2024	3.55	7.94
Nyc	2010	8.18	3.47
Nyc	2011	21.60	0.67
Nyc	2013	6.07	1.68
Nyc	2014	3.42	3.17
Nyc	2015	11.53	7.36
Nyc	2016	6.43	9.95
Nyc	2017	7.93	7.65
Nyc	2018	6.49	0.02
Nyc	2019	6.28	3.70
Nyc	2021	32.29	8.18
Nyc	2022	7.33	5.70
Nyc	2023	23.58	17.40
Nyc	2024	3.71	1.43
Tokyo	2010	18.64	26.78
Tokyo	2011	15.30	23.93
Tokyo	2012	10.00	34.07
Tokyo	2013	3.56	15.83
Tokyo	2014	13.38	27.39
Tokyo	2015	18.84	35.21
Tokyo	2016	6.85	32.05
Tokyo	2017	5.09	18.42

Tokyo	2018	10.16	24.40
Tokyo	2019	11.41	43.75
Tokyo	2021	2.12	11.92
Tokyo	2022	1.97	8.88
Tokyo	2023	7.16	31.76
Tokyo	2024	4.70	17.47

Note: Method 2 race-window pollution values represent the mean NO_2 and $PM_{2.5}$ concentration during a 3-hour window centred on race start time, derived from ERA5-based atmospheric reanalysis data. NO_2 = nitrogen dioxide; $PM_{2.5}$ = particulate matter ≤2.5 µm. SD = standard deviation.

Supplementary Material — Additions for Revised Manuscript

Manuscript: Air Pollution Impairs Marathon Performance: A Cross-Sectional Analysis of 2.7 Million Finishers Across Six World Marathon Majors

Authors: James A. Donaldson; Samuel Cai; Joshua Vande Hey; Anna Hansell; Thomas Yates; André Ng; Jamie O'Driscoll

This file collects the new and corrected supplementary content prepared during revision. The numbered Supplementary Tables below are intended to be merged into the single combined Supplementary PDF (see assembly recipe in revision/REVISION_PLAN.md). All values were generated from the final Method 2 (3-hour race-window CAMS) analysis and verified against analysis/FINAL_NUMBERS.md.

Supplementary Table S7. Timing basis by marathon series (Reviewer 2, chip vs gun times)

All finishing times analysed are net (chip) times. Each official results platform reports net finishing times, which were the values extracted by our web-scraping scripts.

Marathon series	Results source	Time basis analysed	Race-years included
Berlin	Official BMW Berlin-Marathon results portal	Net (chip)	2010–2024 (excl. 2020)
Boston	Boston Athletic Association results portal	Net (chip)	2010–2024 (excl. 2020)
Chicago	Bank of America Chicago Marathon results portal	Net (chip)	2010–2024 (excl. 2011, 2020)
London	TCS London Marathon results portal	Net (chip)	2010–2024 (excl. 2020 mass event)
New York City	TCS New York City Marathon results portal	Net (chip)	2010–2024 (excl. 2012 [Hurricane Sandy], 2020)
Tokyo	Tokyo Marathon results portal	Net (chip)	2015–2024 (excl. 2020–2022)

Note: race-year availability reflects event cancellations (2020 COVID-19; New York City 2012, Hurricane Sandy) and the years for which comparable individual results were publicly available. The 2013 Boston Marathon was retained in the primary analysis and examined in a sensitivity analysis (Supplementary Table S5).

Supplementary Table S8. NO_2 effects expressed as a percentage of mean finishing time (Reviewer 1)

Effects are from sex-, performance-, and age-stratified single-pollutant linear mixed models, expressed both in absolute terms (minutes per 1 μg/m³ NO_2) and as a percentage of the relevant subgroup's mean finishing time.

By sex

Sex	Effect (min per μg/m³)	Mean finish (min)	Effect (% of finish)
Men	1.43	255.4	0.56%
Women	1.56	283.0	0.55%

The absolute effect appears larger in women (ratio 0.92 men:women), but the proportional effect is near-identical (ratio 1.02), indicating that the apparent sex difference reflects women's longer mean finishing times rather than greater proportional susceptibility.

By performance level

Performance group	Effect (min per μg/m³)	Mean finish (min)	Effect (% of finish)
Elite (top 10%)	0.34	186.8	0.18%
Sub-Elite (10–25%)	0.80	215.8	0.37%
Upper Mid (25–50%)	1.32	243.8	0.54%
Mid-Pack (50–75%)	1.83	278.1	0.66%
Recreational (75–90%)	2.12	316.5	0.67%
Back of Pack (90–100%)	2.23	368.5	0.60%

By age group (men)

Age group	Effect (min per μg/m³)	Mean finish (min)	Effect (% of finish)
18–29	2.17	251.0	0.87%
30–39	1.53	247.5	0.62%
40–49	1.38	251.1	0.55%
50–59	1.26	263.9	0.48%
≥60	1.32	291.7	0.45%

The performance and age gradients persist on the relative scale, confirming genuinely larger proportional effects in slower and younger runners.

Supplementary Table S5 (addition). Sensitivity analysis excluding the 2013 Boston Marathon

Single- and two-pollutant linear mixed models re-estimated after excluding the 2013 Boston Marathon (halted before all participants finished). 95% confidence intervals are estimate ± 1.96 × standard error.

Model	Sex	Full dataset (min per μg/m³) [95% CI]	Excl. Boston 2013 (min per μg/m³) [95% CI]
NO_2 (single)	Men	1.43 [0.66, 2.21]	1.47 [0.74, 2.20]
NO_2 (single)	Women	1.56 [0.63, 2.48]	1.60 [0.73, 2.47]
$PM_{2.5}$ (single)	Men	−0.13 [−0.67, 0.40]	−0.03 [−0.55, 0.49]
$PM_{2.5}$ (single)	Women	−0.14 [−0.76, 0.49]	−0.00 [−0.61, 0.60]

Model	Sex	Full dataset (min per μg/m³) [95% CI]	Excl. Boston 2013 (min per μg/m³) [95% CI]
$PM_{2.5}$ (two-pollutant)	Men	−0.66 [−1.17, −0.15]	−0.55 [−1.04, −0.06]
$PM_{2.5}$ (two-pollutant)	Women	−0.71 [−1.32, −0.09]	−0.56 [−1.16, 0.03]

Excluding Boston 2013 does not materially change the NO_2 association or the null single-pollutant $PM_{2.5}$ association; the negative two-pollutant $PM_{2.5}$ coefficient is attenuated and loses statistical significance in women.

Supplementary Section. Validation of CAMS reanalysis against ground monitoring stations (Reviewer 2)

Methods

To assess the validity of CAMS race-window estimates, we compared CAMS-derived race-window (approximately 06:00–18:00 local time) NO_2 and $PM_{2.5}$ concentrations against contemporaneous ground-level monitoring data for five of the six cities. Monitoring data were obtained from the US Environmental Protection Agency Air Quality System (EPA AQS) for Boston, Chicago and New York City; the UK Department for Environment, Food and Rural Affairs Automatic Urban and Rural Network (DEFRA AURN) for London; and the Berlin Air Quality Monitoring Network (Berliner Luftgütemessnetz, BLUME) for Berlin. Tokyo was excluded as comparable public monitoring data were not available. For each city, 3–5 stations near the marathon course were used, and race-window means were computed across stations with at least three valid hourly measurements. Agreement was summarised using mean absolute error (MAE), mean bias (CAMS minus monitor), root mean square error (RMSE), and Pearson and Spearman correlations.

Supplementary Table S9. Agreement between CAMS reanalysis and ground monitoring stations

Pollutant	City	N race-years	MAE (μg/m³)	Mean bias (μg/m³)	RMSE (μg/m³)	Pearson r	Spearman ρ
NO_2	Berlin	14	11.0	−11.0	12.4	0.64	0.60
NO_2	Boston	14	9.7	−9.7	10.7	0.52	0.56
NO_2	Chicago	14	4.1	+2.2	6.5	0.75	0.56
NO_2	London	14	40.0	−40.0	44.5	0.78	0.85
NO_2	New York City	12	7.0	−7.0	10.8	0.82	0.89
$PM_{2.5}$	Berlin	7	4.0	−2.1	5.6	0.79	0.86
$PM_{2.5}$	Boston	10	12.0	+12.0	15.5	0.09	0.26
$PM_{2.5}$	Chicago	7	5.3	+3.3	7.8	0.34	0.64

Pollutant	City	N race-years	MAE (μg/m³)	Mean bias (μg/m³)	RMSE (μg/m³)	Pearson r	Spearman ρ
$PM_{2.5}$	London	13	6.1	+5.3	9.0	0.80	0.68
$PM_{2.5}$	New York City	10	3.4	−0.7	4.3	0.75	0.44

Mean bias = CAMS minus monitor (negative = CAMS underestimates). Monitor values represent the mean across 3–5 stations along the marathon course. London monitoring stations include kerbside sites (e.g. Marylebone Road), which explains the large negative NO_2 bias relative to the gridded CAMS background estimate.

Results

For NO_2, CAMS showed moderate-to-strong rank-order agreement with ground monitors in all five cities (Pearson r = 0.52–0.82), while systematically underestimating absolute concentrations relative to (frequently kerbside) monitoring stations. $PM_{2.5}$ agreement was strong in Berlin, London and New York City (r = 0.75–0.80) but weak in Boston (r = 0.09) and Chicago (r = 0.34). Because exposure enters the regression models through between-event contrasts, the strong rank-order agreement for NO_2 supports the use of CAMS as a valid index of relative race-day exposure. The weaker measurement performance for $PM_{2.5}$ in two cities is consistent with greater exposure measurement error for $PM_{2.5}$, which is relevant to interpreting the negative $PM_{2.5}$ coefficient in the two-pollutant models (Discussion).
